# The molecular mechanism of acute liver injury and inflammatory response induced by Concanavalin A

**DOI:** 10.1186/s43556-021-00049-w

**Published:** 2021-08-10

**Authors:** Xiaoxiao Liu, Ting Yu, Yuzhu Hu, Longzhen Zhang, Junnian Zheng, Xiawei Wei

**Affiliations:** 1 Laboratory of Aging Research and Cancer Drug Target, State Key Laboratory of Biotherapy and Cancer Center, National Clinical Research Center for Geriatrics, West China Hospital, Sichuan University, Chengdu, China; 2 Department of Radiation Oncology, Cancer Center, Affiliated Hospital of Xuzhou Medical University, Jiangsu Center for the Collaboration and Innovation of Cancer Biotherapy, Cancer Institute, Xuzhou Medical University, Xuzhou, 221000 China; 3 Department of Pathology, West China Hospital, Sichuan University, Chengdu, China; 4 Department of Medical Oncology, Cancer Center, West China Hospital, Sichuan University, Chengdu, China

**Keywords:** Liver damage, Mixed lineage kinase domain like protein, Necroptosis, Mitochondrial DNA, Liver inflammation

## Abstract

Acute liver injury is a common but urgent clinical condition, and its underlying mechanism remains to be further elucidated. Concanavalin A (ConA)-induced liver injury was investigated in the study. Different from the caspase-dependent cell apoptosis in lipopolysaccharide/D-aminogalactose (LPS/D-GalN) induced liver injury, ConA-induced hepatocyte death was independent on caspase. Increased hepatocytic expressions of mixed lineage kinase domain like (MLKL) and receptor-interacting protein kinase 1 (RIPK1), and higher serum concentration of tumor necrosis factor-α (TNF-α) were noticed in mice with ConA-induced liver injury. Inhibition of RIPK1 protein or deletion of MLKL gene could significantly attenuate the acute liver injury and improve mice survival. Besides, the ConA treatment induced severe hepatic inflammation in wide type (*WT*) mice in comparison with *Mlkl*^*−/−*^ mice, suggesting the RIPK1-MLKL-mediated hepatocellular necroptosis might participate in the process of liver injury. Moreover, mitochondrial damage associated molecular patterns (DAMPs) were subsequently released after the hepatocyte death, and further activated the p38 mitogen-activated protein kinase (MAPK) pathway, which could be reduced by deletion or inhibition of Toll-like receptor 9 (TLR9). Taken together, our research revealed that ConA-induced acute liver injury was closely related to TNF-α-mediated cell necroptosis, and inhibiting RIPK1 or deleting MLKL gene could alleviate liver injury in mice. The mitochondrial DNA released by dead hepatocytes further activated neutrophils through TLR9, thus resulting in the exacerbation of liver injury.

**Supplementary Information:**

The online version contains supplementary material available at 10.1186/s43556-021-00049-w.

## Introduction

As the largest solid organ of human body, liver involves in the synthesis or metabolism of various substances, including carbohydrate, fat, protein, vitamins and some harmful substances. Normal functioning of the liver is essential for maintaining body homeostasis. Liver injury caused by viruses, autoimmune responses, alcohol, drugs or other factors is a serious threat to human health [1–4]. Clinically, patients diagnosed with acute liver injury often have poor prognosis and high fatality rate due to the acute onset, severe symptoms and concomitant organ failure [5, 6]. Although liver transplantation is recognized as the optimal choice for treating acute liver injury, its clinical application is restricted due to the lack of suitable donor liver, high costs and subsequent long-term drug-related side effects [7, 8]. Hence, it is valuable to investigate the underlying mechanisms of acute liver injury and seek for new therapeutic approach.

As a T cell polyclonal mitogen, Concanavalin A (ConA) could be accumulated in liver and cause liver-specific acute injury by activating T cells. Moreover, auto-reactive T cells and auto-antibodies are commonly observed in autoimmune hepatitis (AIH). The T cell-dependent acute liver injury mice model mediated by ConA was successfully established by Tiges in 1992, which was manifested with acute onset, rapidly increased serum levels of glutamic oxalacetic transaminase (AST) and glutamic-pyruvic transaminase (ALT), liver congestion and large necrosis area under the microscope [9]. Owing to the autoimmune-related feature, ConA induced acute liver injury mice model is considered to be an important experimental model of AIH.

It is believed that acute liver injury is related to the death of hepatocytes and release of inflammatory factors. As a new pattern of cell death, necroptosis is different from necrosis and apoptosis [10]. The cell morphology of necroptosis is similar to that of necrosis, both of which are characterized by cell membranes destruction and cellular swelling, while this necroptosis process is programmed and independent of caspases activation [11, 12]. Studies have shown that the necroptosis relies on the activation of tumor necrosis factor-α (TNF-α) and other tumor necrosis factor related apoptosis inducing ligand (TRAIL) receptor [13–15]. Furthermore the receptor-interacting proteins (RIP) and mixed lineage kinase domain like (MLKL) protein have been reported to play crucial parts in the course of necroptosis [16, 17]. There are various RIP kinases (RIPK) subtypes, in which the RIPK1 and RIPK3 could regulate the process of necroptosis [17, 18]. RIPK1 regulates the recruitment of necrosome, which could be blocked by the specific RIPK1 inhibitor necrostatin-1 (Nec-1), thereby inhibiting cellular necroptosis [10, 19]. RIPK3 has been reported not only to enhance the effect of RIPK1, but also to activate downstream MLKL molecular signals together with RIPK1 [20, 21]. When MLKL protein oligomer translocate to plasma membrane, it could cause the sodium ions inflow, cell membrane rupture and eventually lead to cell death [10, 22]. Besides, MLKL protein and necrotic complexes are found to be distributed in mitochondria and mitochondria-related membranes, indicating RIPK-MLKL mediated cell necroptosis might be closely associated with mitochondria [23].

The release of endogenous molecules after cell death can activate innate immunity, known as damage associated molecular patterns (DAMPs) [24]. DAMPs regulate the immune system by affecting the function of dendritic cells, macrophages, eosinophils and neutrophils. After trauma, the mitochondrial DAMPs, including mitochondrial DNA (mtDNA) and mitochondrial formyl peptide, are released into circulation and activate the innate immune system. In recent years, the research on mtDNA has attracted much attention [25]. As the genetic substance carried by mitochondria, mtDNA is different from nuclear DNA, but more similar to bacterial DNA, rich in CpG sequences [26, 27]. Studies found that CpG can stimulate the phosphorylation of mitogen activated protein kinase (MAPK) through Toll-like receptor 9 (TLR9), thereby triggering the release of inflammatory factors [28–30]. Moreover, mitochondrial formyl peptides affect neutrophils function by binding to its receptor on the surface of neutrophils [31]. Therefore, these mitochondrial DAMPs are of importance in activating inflammatory response, while the correlation between acute autoimmune liver injury and mitochondrial DAMPs as well as the underlying mechanisms remain largely unknown.

Herein, the ConA-induced acute liver injury mice model was established to explore underlying mechanisms of acute liver injury and inflammatory responses. We infer that the release of mitochondrial DAMPs after TNF-α related hepatocyte necroptosis is critical for causing inflammatory response in acute auto-immune liver injury and the RIPK-MLKL signal pathway might well be a potential therapeutic target in future clinic.

## Results

### ConA-induced acute live injury was featured with necrotic liver injury

**Fig. 1:**
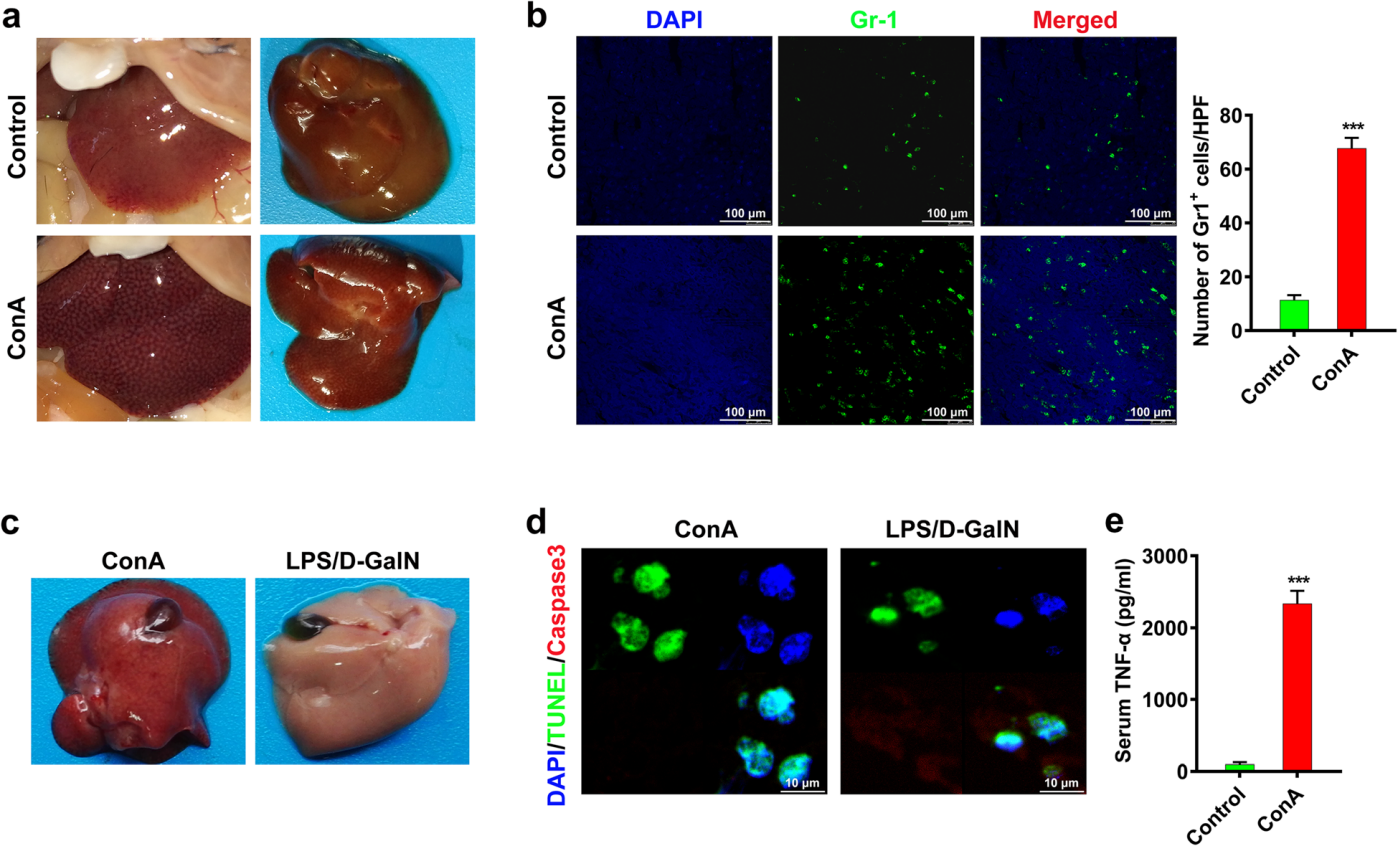
ConA-induced acute live injury was featured by necrotic liver injury. **a** Acute liver damage was induced in *WT* C57BL/6 mice. After 10 h of ConA injection, the pathologic gross picture was presented. **b** The Gr-1 immunofluorescence staining result indicated a large amount of inflammatory cells infiltration in liver tissues induced by ConA. **c** Representative images of mice liver showed that ConA induced liver injury had different appearance from LPS/D-GalN induced liver injury. **d** The liver section of LPS/D-GalN model showed both TUNEL positive and cleaved caspase-3 positive, while the liver section of ConA model was primarily TUNEL positive. **e** The serum TNF-α level was significantly increased at 10 h after ConA injection. (***p < 0.001)

First, the C57BL/6 mice were intravenously injected with ConA (20 mg/kg) for establishing the acute liver injury model. Obvious acute liver injury was observed 10 h after ConA injection (Fig. 1a). The results of Gr-1 immunofluorescence staining showed increased infiltration of inflammatory cells in the liver of ConA-treated mice (Fig. 1b). To clarify the pattern of ConA-induced hepatocyte death, lipopolysaccharide/D-aminogalactose (LPS/D-GalN)-induced liver injury model was established to be a contrast. The hepatocyte death in LPS/D-GalN model are known to be caspase-dependent apoptosis [32]. Intriguingly, the gross pathology in ConA-induced liver injury was significantly different from that in LPS/D-GalN -induced liver injury (Fig. 1c). The liver from ConA model only showed TUNEL positive, different from both positive staining of TUNEL and cleaved caspase-3 in the liver from LPS/D-GalN model which suggested the ConA-induced liver injury tend to be a caspase-independent necroptosis (Fig. 1d). The cells necroptosis depends on the activation of TNF-α, thus TNF-α is thought to be essential for ConA-induced liver injury. To explore the effects of TNF-α in ConA-induced liver injury, the serum level of TNF-α in mice was detected. An obvious increase of TNF-α level was observed in mice serum after ConA injection (Fig. 1e). Moreover, after the intravenous injection of TNF-α protein (100 μg/kg) for 10 h, C57BL/6 mice showed significantly more neutrophils infiltration in liver than control mice (Supplementary Fig. 1). Therefore, different from caspase-dependent hepatocyte apoptosis in LPS/D-GalN induced liver injury, ConA-induced hepatocyte death was independent on caspase, suggesting it might be a TNF-α-mediated necroptosis.

### Role of RIPK1 and RIPK3 in ConA-induced acute liver injury

**Fig. 2:**
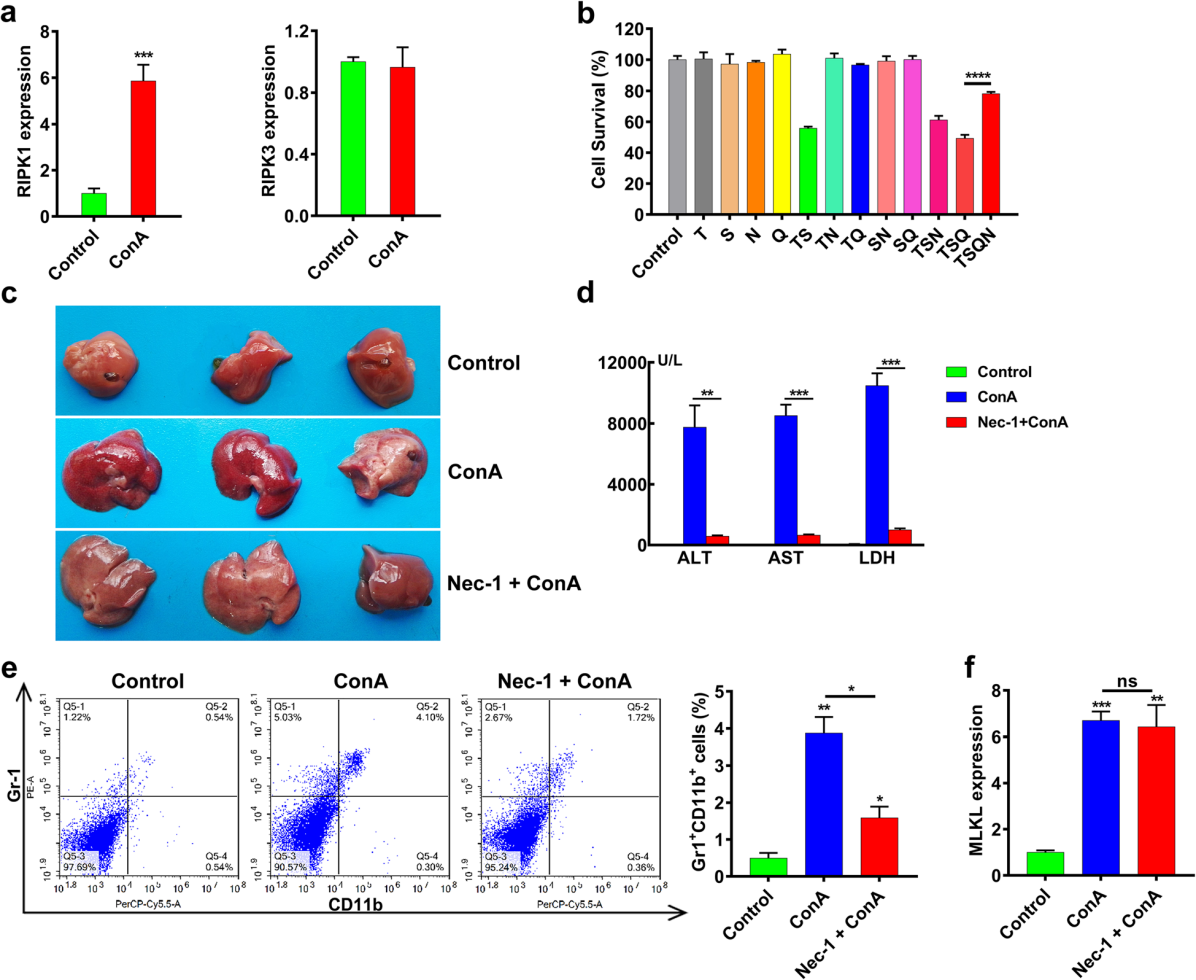
Role of RIPK1 and RIPK3 in ConA-induced acute liver injury. Both in vitro and in vivo studies were conducted to investigate the roles of RIPK1 and RIPK3 in ConA-induced acute liver injury. **a** The RT-PCR analysis showed the intrahepatic mRNA level of RIPK1 was significantly elevated in mice treated with ConA compared with untreated mice, while no significant difference in the intrahepatic mRNA level of RIPK3 between ConA-treated and untreated mice. **b** L929 cells treated with RIPK1 inhibitor Necrostatin-1 (N) were found resistant to the necroptosis induced by the combination of hTNF-α (T), Smac-mimetic (S) and caspase-inhibitor QVD-OPH (Q). **c** The gross images of livers showed of Nec-1 pretreatment alleviated ConA-induced liver injury. **d** The Nec-1 pretreatment attenuated the increase of serum ALT, AST and LDH induced by ConA. **e** The Nec-1 pretreatment reduced the infiltration of Gr1^+^CD11b^+^ cells in the liver of ConA-treated mice. **f** The hepatic expression of MLKL, a key protein in necroptosis, was increased in ConA-treated mice compared to untreated mice. (*p < 0.05, **p < 0.01, ***p < 0.001, ****p < 0.0001)

As a novel class of kinase, RIPK1 is associated with regulating apoptosis and TNF-α induced necroptosis. RIPK, as a homologous kinase of RIPK1, has been regarded as a key regulator in caspase-independent cell death [20]. The intrahepatic mRNA level of RIPK1 was significantly increased after ConA treatment, while no significant change of the RIPK3 expression level was observed (Fig. 2a). To identify whether RIPK1 was involved in hepatocyte necroptosis, the specific RIPK1 inhibitor Necrostatin-1 (Nec-1) were used for determining the ConA cytotoxicity in L929 cells. The L929 cells pretreated with Nec-1 were resistant to the necroptosis induced by hTNF-α (T), Caspase agonist analogue Smac (S) and caspase-inhibitor QVD-OPH (Q) combination (Fig. 2b). Encouraged by the protective effect of Nec-1 in vitro, we further investigated the effect of RIPK1 inhibition in ConA-induced liver injury model. Half an hour before ConA injection, the mice were intravenously administrated with Nec-1 (400 μg/kg), and the livers were collected after 10 h. The Nec-1 pretreatment successfully alleviated the ConA-induced liver injury and reduced serum levels of ALT, AST and LDH (Fig. 2c, d). What’s more, the Nec-1 pretreatment also reduced ConA-induced neutrophilic infiltration in the liver (Fig. 2e). Therefore, the RIPK1 activity but not RIPK3 was relevant to the ConA-induced acute liver injury. We further detected the intrahepatic expression of MLKL, the as yet most known end-stage effector in necroptosis pathway [33]. The injection of ConA significantly increased the intrahepatic expression of MLKL, while pretreatment with Nec-1 showed no significant effect on MLKL expression, indicating MLKL expression was independent of RIPK1 during ConA-induced hepatocyte necroptosis (Fig. 2f).

### MLKL may play a vital role in ConA-induced acute liver injury

**Fig. 3:**
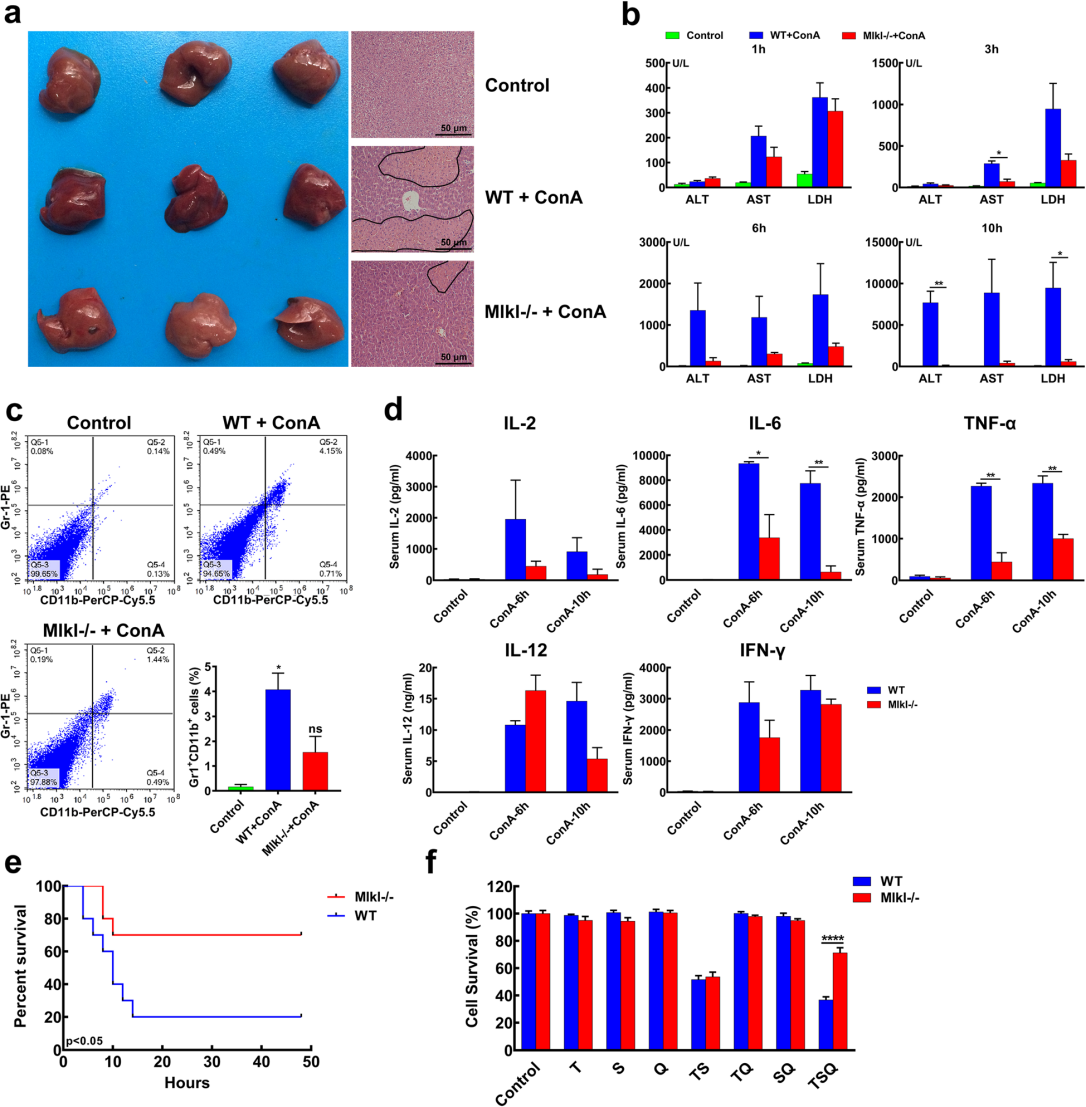
MLKL may play a vital role in ConA-induced acute liver injury. **a** Representative photograph of mice liver from normal group, ConA-treated *WT* group and ConA-treated *Mlkl*^*−/−*^ group, and HE staining of liver sections suggested obviously decreased ConA-induced hepatocyte necrosis in *Mlkl*^*−/−*^ group than *WT* group. **b** Serum levels of ALT, AST and LDH were detected in normal mice, ConA-treated *WT* mice and ConA-treated *Mlkl*^*−/−*^ mice at 1, 3, 6 and 10 h. **c** Gr1^+^CD11b^+^ cells were significantly increased in ConA-treated *WT* mice liver than that in ConA-treated *Mlkl*^*−/−*^ mice liver. **d** The *Mlkl*^*−/−*^ mice showed significantly lower IL-2, IL-6, IL-12, IFN-γ and TNF-α levels than *WT* mice at 6 and 10 h after ConA injection. **e** Survival curve of ConA-treated mice from *WT* group and *Mlkl*^*−/−*^ group (*p < 0.05). **f** Hepatocytes from *Mlkl*^*−/−*^ mice could resist the necroptosis caused by the combination of hTNF-α (T), Caspase agonist analogue Smac (S) and caspase-inhibitor QVD-OPH (Q). (*p < 0.05, **p < 0.01, ****p < 0.0001)

Although necroptosis is known to be mediated by RIP kinases and MLKL, the significance of MLKL in acute liver injury remains unclear [33]. To further explore the effect of MLKL on ConA-induced acute liver injury, the *WT* mice and *Mlkl*^*−/−*^ mice were intravenously administrated with ConA (20 mg/kg) and sacrificed 10 h after injection. Gross and histological results showed siginificantly relieved liver injury in *Mlkl*^*−/−*^ mice in comparison with *WT* mice (Fig. 3a). The ALT, AST and LDH were used to quantify the severity of liver injury. Mice blood samples were collected for serological biochemical analysis at the set time points (1, 3, 6 and 10 h). The results showed the serum levels of ALT, AST and LDH were increased with time, and the liver injury was much more severe in *WT* mice when compared to that in *Mlkl*^*−/−*^ mice (Fig. 3b). The *WT* mice developed severe liver injury (ALT > 1000 U/L) at both 6 and 10 h after ConA injection, while *Mlkl*^*−/−*^ mice suffered mild liver injury. According to TUNEL assay, hepatocyte apoptosis mediated by ConA was obviously reduced in *Mlkl*^*−/−*^ mice compared with that in *WT* mice (Supplementary Fig. 2). Furthermore, the hepatic infiltration of neutrophils was also significantly reduced in *Mlkl*^*−/−*^ mice in comparison with that in *WT* mice (Fig. 3c), accompanied by significantly lower serum levels of IL-2, IL-6, IL-12, IFN-γ and TNF-α (Fig. 3d). Survival analysis also demonstrated a significantly improved survival of *Mlkl*^*−/−*^ mice in comparison with *WT* mice after the ConA injection (Fig. 3e). Moreover, primary isolated hepatocytes from *WT* mice and *Mlkl*^*−/−*^ mice were used to explore the role of Mlkl in hepatocyte death. As shown in Fig. 3f, hepatocytes from *Mlkl*^*−/−*^ mice were resistant to the necroptosis induced by combination of hTNF-α (T), Caspase agonist analogue Smac (S) and caspase-inhibitor QVD-OPH (Q). In short conclusion, these results indicated the deficiency of MLKL gene might play a protective role in ConA-induced acute liver injury.

### ConA-induced acute liver injury in mice was mediated by mtDNA release

**Fig. 4:**
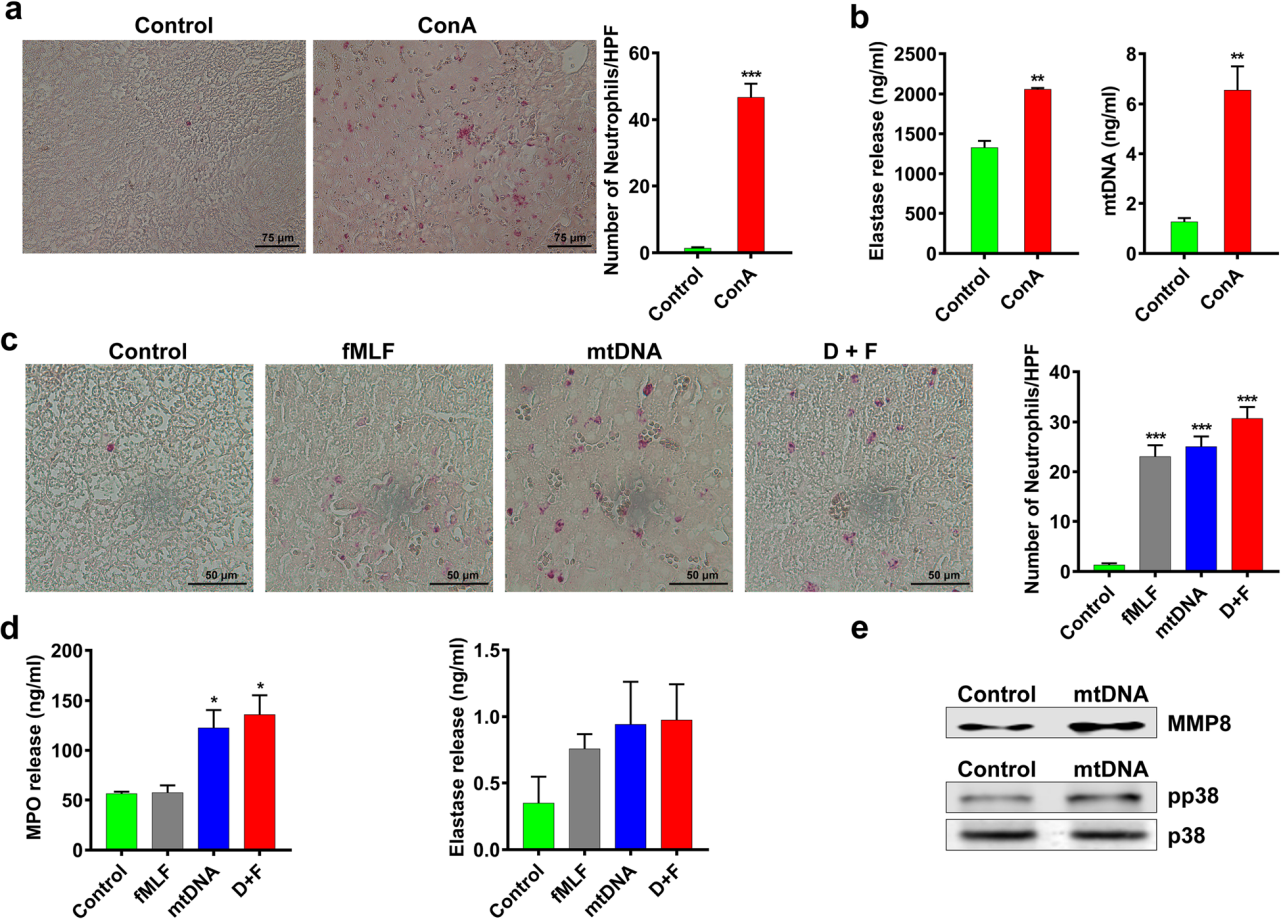
ConA-induced acute liver injury in mice was mediated by mtDNA release after cell death. **a** A large number of esterase-positive neutrophils infiltrated in mice liver after ConA injection. **b** The serum level of elastase and mtDNA were increased after ConA injection. **c** The injection of fMLF, mtDNA, and D + F (mtDNA+fMLF) induced obvious neutrophils infiltration in mice liver. **d** The mtDNA and D + F induced MPO and elastase to release from neutrophils, while the fMLF showed a mild effect. **e** The mtDNA promoted the secretion of matrix metalloproteinase-8 (MMP-8) from neutrophils and caused the activation of p38-MAPK pathway. (*p < 0.05, **p < 0.01, ***p < 0.001)

The hepatocyte necroptosis induced by ConA was postulated to trigger subsequent liver inflammation. Significantly increased infiltration of esterase-positive neutrophils was observed in the liver of ConA-treated mice (Fig. 4a). The serum levels of elastase and mtDNA were also increased after ConA injection (Fig. 4b). Moreover, the serum level of mtDNA was obviously lower in *Mlkl*^*−/−*^ mice in comparison with that in *WT* mice after ConA treatment (Supplementary Fig. 3a). The ConA treatment also significantly promoted the elastase release in *WT* mice, while no obvious increase in elastase release was observed in *Mlkl*^*−/−*^ mice (Supplementary Fig. 3b). The release of endogenous DAMPs has been speculated to be one possible explanation for the necrotic cells caused inflammatory response. Intracellular components including mitochondria could be served as danger signal to alert the innate immune system [34]. Thereafter, we supposed the mitochondrial leakage from necrotic cells might play a crucial part in ConA-induced acute liver injury. Ten hours after being intravenously injected with the synthetic peptide N-formyl-Met-Leu-Phe (fMLF) or extracted mtDNA, the mice were sacrificed. The result showed fMLF, mtDNA, and D + F (mtDNA + fMLF) all induced neutrophil to aggregate in the liver (Fig. 4c). Myeloperoxidase (MPO), a maker of antimicrobial activity, is most abundantly expressed in neutrophil, and the elastase secreted by neutrophils also has important immune functions. We observed that mtDNA could induce the release of MPO and elastase from neutrophils, while fMLF exhibited a mild effect (Fig. 4d). Moreover, the mtDNA stimulated the secretion of matrix metalloproteinase-8 (MMP8) and promoted the phosphorylation of p38 protein in neutrophils, suggesting the activation of the p38 mitogen-activated protein kinase (MAPK) pathway (Fig. 4e). Taken together, the leakage of mitochondrial components from necrotic cells, especially mtDNA, could activate neutrophils through p38-MAPK pathway, stimulate the release of MPO and elastase, and thus evoking the inflammatory response.

### ConA could induce inflammatory responses through TLR9 pathway

**Fig. 5:**
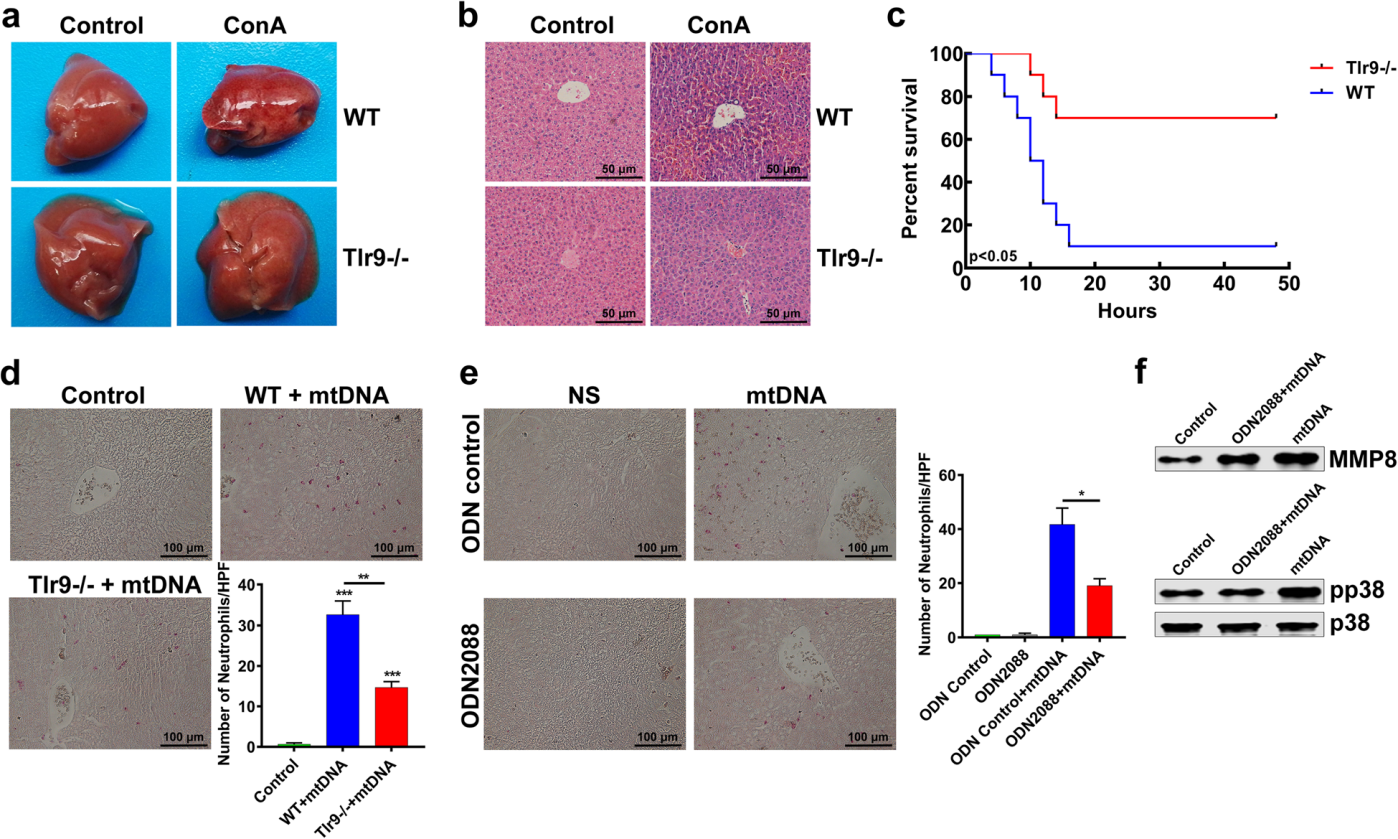
ConA induced hepatic inflammation through TLR9 pathway. **a** The liver injury was less severe in *Tlr-9*^*−/−*^ mice after ConA injection. **b** The HE staining result of liver sections suggested less hepatocytes death in *Tlr-9*^*−/−*^ mice than that in *WT* mice after ConA injection. **c** The survival time of *Tlr-9*^*−/−*^ mice treated with ConA was significantly longer than that of *WT* mice treated with ConA. **d** Significantly decreased infiltration of esterase-positive neutrophils was observed in the liver of mtDNA-treated *Tlr9*^*−/−*^ mice, in comparison with mtDNA-treated *WT* mice. **e** The *WT* mice pretreated with ODN2088 had less neutrophils infiltration in liver after mtDNA injection. **f** ODN2088 suppressed the mtDNA-induced secretion of MMP8 and the activation of p38-MAPK pathway in neutrophils. (*p < 0.05, **p < 0.01, ***p < 0.001)

Since the preliminary study found that mtDNA might play a role in ConA-induced hepatitis, we tried to figure out the pathway by which the mtDNA played a role. Phosphorylation of MAPK has been reported to trigger subsequent inflammatory responses through TLR9 pathway [35]. Thus, we discussed the role of TLR9 pathway in ConA-induced hepatic inflammation. Both gross and histological observations showed that the liver injury of *Tlr-9*^*−/−*^ mice treated with ConA was less than that of *WT* mice treated with ConA (Fig. 5a, b). The serological biochemical analysis confirmed that liver injury was alleviated in *Tlr-9*^*−/−*^ mice, with significantly lower levels of liver damage markers (ALT, AST and LDH) (Supplementary Fig. 4a). The survival rate of ConA-treated *Tlr-9*^*−/−*^ mice was significantly improved as well (Fig. 5c). After intravenous injection of mtDNA into *WT* mice and *Tlr9*^*−/−*^ mice, a significant reduction in esterase-positive cells infiltration was observed in *Tlr9*^*−/−*^ liver sections (Fig. 5d). Moreover, the result of pretreatment with ODN2088, a TLR-9 antagonist, was in consistence with the above finding, that is, pretreatment with ODN2088 reduced neutrophil infiltration in *WT* mice (Fig. 5e). Additionally, the treatment of ODN2088 also inhibited the secretion of MMP8 from neutrophils and mtDNA-induced activation of p38-MAPK pathway (Fig. 5f). Therefore, mtDNA can activate neutrophils through TLR-9 pathway. The STING signal pathway has been known to be distinguished from other DNA sensing pathways like TLR9 pathway [36]. Thus, we further investigated whether STING pathway was involved in ConA-induced liver injury. The results showed that ConA still caused severe liver injury in *Sting*^*−/−*^ mice. Serum biochemical markers showed no significant relief of liver injury and the mice survival was not prolonged (Supplementary Fig. 4b, c and d). These results suggested that the subsequent hepatic inflammation caused by ConA might be attributed to the mitochondrial leakage from necrotic hepatocytes, which activate p38-MAPK pathway in neutrophils through TLR-9 pathway.

## Discussion

Acute liver injury is a common but urgent clinical condition, because fulminant liver failure may occur after the exacerbation of acute liver injury and lead to unfavorable prognosis. The underlying molecular mechanisms of acute liver injury remain largely unknown. A deeper understanding of the specific mechanisms will provide strategies to develop more efficient treatment to acute liver injury. The ConA-induced T cell mediated acute liver injury model is an important experiment model of acute liver injury, which is often applied to investigate the pathogenesis of viral hepatitis and autoimmune hepatitis [37]. Herein, we attempt to investigate and explain the potential mechanisms of ConA-induced acute liver injury.

This study revealed that RIPK1-MLKL is involved in the TNF-α related cell death pathway and plays a critical role in ConA-induced liver injury. The hepatic expression of MLKL was significantly increased in mice treated with ConA, while the deficiency of MLKL gene alleviated the liver injury and improved the survival of mice. Our results found the cell apoptosis in ConA induced liver injury was independent on caspase pathway, which was different from caspase dependent cell apoptosis in LPS/D-GalN induced liver injury. Moreover, the serum level of TNF-α was substantially increased in ConA-treated mice, which was capable of inducing both cell apoptosis and necroptosis. As an important component in necroptosis, the hepatic expression of RIPK1 was increased in ConA-treated mice and the inhibition of RIPK1 had a protective effect on liver injury. Moreover, RIPK1 inhibition or MLKL deficiency reduced cell death caused by the combination of hTNF-α, caspase-activator analogue Smac and caspase-inhibitor QVD-OPH in vitro, suggesting RIPK1 and MLKL participated in TNF-α related cell death in acute liver injury. Furthermore, the mtDNA was found to release extracellular, and resulted in neutrophils activation and pro-inflammatory factors secretion. The ablation of TLR9 not only attenuated hepatic inflammation and alleviated ConA induced liver injury, but also improved the survival of mice.

Plenty of evidence has confirmed the involvement of TNF-α pathway in liver injury process [38, 39]. Herein, we reported the TNF-α-related necroptosis which was mediated by RIPK1 and MLKL contributed to ConA induced acute liver injury. TNF-α has been disclosed to show dichotomous role in cell death process, triggering caspase-8 dependent apoptosis or RIPK1 dependent necroptosis [40]. The cell death would switch to necroptosis rather than apoptosis when deubiquitinated RIPK1 presented with caspase unactivated [41]. Our results showed that cells in liver sections were TUNEL positive but cleaved caspase-3 negative, indicating this kind of cell death was more likely to be necroptosis. One previous study has reported that the liver injury induced by ConA could not be attenuated by caspase inhibition, which supported our hypothesis. The blockage of necroptosis has been demonstrated to ameliorate tissue damage in atherosclerosis, pancreatitis and systemic inflammatory response [42, 43]. What’s more, study has found RIPK3 or MLKL deficiency partially alleviated liver inflammation in TNF-α-dependent multi-organ inflammation that caused by *Sharpin*-deficiency [44]. In this study, increased hepatic expressions of MLKL and RIPK1 were observed and MLKL deficiency or RIPK1 inhibition exhibited protective roles in acute liver injury process. However, no significant change about the expression level of RIPK3 in liver tissue was observed, which might be owing to intrinsically weak hepatic expression of RIPK3 [45]. All above evidences supported that RIPK1-MLKL mediated cell necroptosis could contribute to ConA induced acute liver injury.

Mitochondrial DAMPs was disclosed to activate innate immunity and cause sterile inflammatory response [46]. Mitochondrial DAMPs in circulation could arouse organ injury mediated by neutrophils [47]. Our previous study has revealed that mitochondrial DAMPs could be released after cell death and triggering pulmonary inflammation through activating neutrophils [48]. Results of this study showed increased hepatic infiltration of neutrophils and increased mtDNA content in mice serum after treating with ConA, thereupon it was inferred that the release of mitochondrial DAMPs after cell necroptosis could partially drive hepatic inflammation. In consistence, further results showed the hepatic mtDNA and fMLF could induce infiltration of neutrophils and improved secretions of MPO, elastase and MMP-8 as well as promoted the phosphorylation of P38. mtDNA recognized by TLR9 was able to trigger inflammatory response and release pro-inflammatory factors including TNF-α or IL-6 or adhesion molecules [49]. Research found genetic ablation of TLR9 reduced idiopathic liver injury, fibrosis and hepatocellular carcinoma (HCC) in hepatic deletion of TGF-β-activated kinase 1 (Tak1ΔHep) mice [50]. We revealed the deficiency or inhibition of TLR9 could mitigate hepatic inflammation and restrain the activation of neutrophils, thus reducing the release of inflammatory mediators. TLR9 deficiency successfully abated ConA induced hepatic injury and improved the survival of mice.

In conclusion, we proposed that TNF-α-related cell death pathway involved in ConA-induced acute liver injury. Hepatocellular necroptosis contributed to the hepatoxicity induced by ConA. Inhibiting RIPK1 protein or deleting MLKL gene could significantly attenuate acute liver injury in mice. What’s more, the mitochondrial DAMPs released after cell death could activate neutrophils p38 MAPK pathway through TLR9 pathway, thus promoting pro-inflammatory factors release and resulting in the exacerbation of liver injury. The association between RIPK1 and RIPK3 expression and the relationship of RIPK1-MLKL pathway activation and mtDNA release remain to be elucidated in our future studies. In this contribution, the potential molecular mechanism of acute liver injury and inflammatory response in vivo were elucidated, and our study might provide possible target for clinical treatment of acute liver injury especially autoimmune acute liver injury.

## Materials and methods

### Animals

6–8 weeks old Female C57BL/6 mice were purchased from Beijing Huafukang Biotechnology Co. Ltd. (Beijing, China). The *Mlkl*^*−/−*^ mice and *Tlr9*^*−/−*^ mice were provided by the State Key Laboratory of Biotherapy (SKLB). All animal experiments in this study were approved by the Animal Experimental Ethics Committee of SKLB, Sichuan University (Chengdu, China) and conducted according to the guidelines for the care and use of laboratory animals.

### Neutrophils extraction

Healthy 6–8 weeks old wild type (*WT*) female C57BL/6 mice were sacrificed and immersed into 75% ethanol solution for sterilization. The tibia and femur of mice were separated under sterile conditions, and Serum-free RPMI 1640 culture medium was used to flush bone marrow cavity with a 1 ml syringe until bone marrow cavity became white. Gradient separation solution and filtered cells suspension were added into the centrifuge tube in sequence, followed by gradient centrifugation for 0.5 h at 800 g. Then, the middle layer of cells between two separation solutions was gently pipetted and re-suspended with RPMI 1640 medium containing 10% FBS for later use. The purity of the extracted neutrophils was about 90%.

### Western blot assay

Cells were collected and lysed with 1 ml Lysis Buffer containing phosphatase inhibitor, protease inhibitor and phenylmethanesulfonyl fluoride (PMSF). The supernatant was quantitatively analyzed and prepared for SDS-PAGE electrophoresis. For analyzing proteins in cell culture supernatant, the supernatant was concentrated by ultrafiltration method. Next, samples were separated by electrophoresis and transferred to PVDF membrane. After blocked by skim milk, the membrane was sequentially incubated with primary antibody (1:1000, Cell Signaling Technology, USA) and secondary antibody (1:5000, Beyotime, China). Finally, the chemiluminescence reagent was used for protein band visualization.

### MTT assay

L929 cells and primary hepatocytes were separately seeded into 96-well plates (5000 cells/well) and cultivated overnight. Then, cells were treated with different agents (hTNF-α: 50 ng/ml (T), Smac-mimetic (S): 500 nM, Necrostatin-1 (N): 50 μM, QVD-OPH (Q): 5 μM) for 18 h. Then, 20 μl MTT solution (5 mg/ml) was added to each well and incubated for 3 h. The culture supernatant was removed and 150 μl DMSO was added to each well. The absorbance was measured at 570 nm wavelength by a microplate reader.

### Flow cytometry analysis

Liver tissue were cut into slices and suspended in serum-free RPMI1640 culture medium. The tissue suspension was filtered by cell strainer and processed with erythrocyte lysis buffer. The obtained cells were washed with phosphate buffer solution (PBS) and then stained with Gr1 and CD11b fluorescent antibodies for 0.5 h at 4 °C. Stained cells were re-suspended by PBS. The stained samples were detected by a flow cytometer (Accuri™ C6, BD, USA).

### Serum biochemical tests and ELISA assay

Mice serum was obtained and analyzed for ALT, AST and LDH levels by a biochemical analyzer (Hitachi, Tokyo, Japan). All ELISA procedures were performed referring to the manufacture descriptions (mouse IL2, IL6, IL-12, IFN-γ and TNF-α ΕLISA detection kits from Ebioscience, USA, neutrophil elastase ELISA kits from Cusabio, China and myeloperoxidase (MPO) mouse ELISA kit from Invitrogen, USA). The concentrations of different cytokines in samples were calculated according to their respective standard curves.

### Hepatocyte mitochondrial DNA extraction

Mice were sacrificed and sterilized in 75% ethanol solution. The liver of mice was isolated and homogenized on ice under sterile condition. Next, the obtained hepatic tissue slurry was processed according to mitochondrial DNA isolation kit (Abcam, Cambridge, UK). The obtained mitochondrial DNA samples was then quantified and stored for further use.

### Acute liver injury model establishment

The mice model of acute liver injury was established by tail vein injection of sterilized PBS that containing 2.5 mg/ml ConA at a dose of 20 mg/kg.

### Immunofluorescence staining of Gr1

The frozen liver tissue was cut into slices and fixed with pre-cooled acetone at 4 °C for 20 mins. The sections were treated with 0.5% triton-X100 for 30 min and sealed with goat serum for 0.5 h. Then Gr1 antibody (1:50, BD, USA) was added and incubated at 4 °C in a wet chamber overnight. Next, the sections were incubated with fluorochrome-conjugated secondary antibody at 37 °C for 1 h. DAPI was applied to label the cell nuclei. The stained liver sections were observed under a fluorescence microscope.

### TUNEL assay

Liver tissues were prepared as paraffin-embedded sections. The TUNEL staining kit (Promega, WI, USA) was used to detect cell apoptosis. Briefly, the tissue sections were baked at 65 °C for 2 h and immersed in xylene, gradient ethanol solution (100%, 95%, 85%, 70%, 50%) and 0.85% NaCl solution. Then, 20 μg/ml protease K was added and incubated for 8 mins. After fixation with 4% paraformaldehyde solution, the equilibrium buffer was added to each tissue section for 5–10 min at room temperature. Next, the reaction mixture containing TdT was added to incubate for 1 h. At last, all sections were immersed in stop reaction solution for 15 mins and washed by PBS. DAPI was used to label the cell nuclei. The stained liver sections were examined under a fluorescence microscope.

### Neutrophil specific esterase staining

The paraffin embedded liver sections were immersed with xylene and gradient ethanol solution (100%, 95%, 85%, 70%, 50%). The neutrophil specific esterase staining was performed according to manufacture instruction of mouse neutrophil specific esterase staining kit (Sigma, MO, USA). The stained liver sections were examined under a light microscope.

### Statistical analysis

All experimental data analysis was performed by SPSS 17.0 software (SPSS, Inc., Chicago, IL, USA). Data were represented by mean ± standard error of mean (SEM). Comparison between two groups was analyzed by Student’s *t* test. *P* value of less than 0.05 was considered to be statistically significant.

## Supplementary Information

**Additional file 1: Supplementary Fig. 1** Gr1^+^CD11b^+^ cells infiltration in TNF-α treated mice and untreated mice. **Supplementary Fig. 2** TUNEL staining of liver sections in *Mlkl*^*−/−*^ mice and *WT* mice treated by ConA. **Supplementary Fig. 3** a The serum level of mtDNA and the elastase release in *WT* mice and *Mlkl*^*−/−*^ mice treated by ConA. **Supplementary Fig. 4** a The serum concentration of ALT, AST and LDH in *Tlr-9*^*−/−*^ mice and *WT* mice after ConA injection. b Gross appearance and the HE staining result of liver section in *Sting*^*−/−*^ mice and *WT* mice after ConA injection. c Serum level of ALT, AST and LDH in *Sting*^*−/−*^ mice and *WT* mice after ConA injection. d The survival time of *Sting*^*−/−*^ mice and *WT* mice treated with ConA.

## Data Availability

All data generated or used during this study appear in the submitted article and its supplementary files.
