# Supplementary material for: The molecular mechanism of acute liver injury and inflammatory response induced by Concanavalin A
Source: Mol Biomed. 2021 Aug 10;2:24. doi: 10.1186/s43556-021-00049-w (PMC8607380; doi:10.1186/s43556-021-00049-w)
Supplement: Supplementary file 1 — Additional file 1: Supplementary Fig. 1 Gr1+CD11b+ cells infiltration in TNF-α treated mice and untreated mice. Supplementary Fig. 2 TUNEL staining of liver sections in Mlkl−/− mice and WT mice treated by ConA. Supplementary Fig. 3 a The serum level of mtDNA and the elastase release in WT mice and Mlkl−/− mice treated by ConA. Supplementary Fig. 4 a The serum concentration of ALT, AST and LDH in Tlr-9−/− mice and WT mice after ConA injection. b Gross appearance and the HE staining result of liver section in Sting−/− mice and WT mice after ConA injection. c Serum level of ALT, AST and LDH in Sting−/− mice and WT mice after ConA injection. d The survival time of Sting−/− mice and WT mice treated with ConA. [file 43556_2021_49_MOESM1_ESM.doc]

## The Molecular Mechanism of Acute Liver Injury and Inflammatory Response induced by Concanavalin A

Xiaoxiao Liu^1,2, #^, Ting Yu^1,3, #^, Yuzhu Hu^1,4, #^, Longzhen Zhang^2^, Junnian Zheng^2^, Xiawei Wei^1,*^

1. Laboratory of Aging Research and Cancer Drug Target, State Key Laboratory of Biotherapy and Cancer Center, National Clinical Research Center for Geriatrics, West China Hospital, Sichuan University, Chengdu, China
2. Department of Radiation Oncology, Cancer Center, Affiliated Hospital of Xuzhou Medical University; Jiangsu Center for the Collaboration and Innovation of Cancer Biotherapy, Cancer Institute, Xuzhou Medical University, Xuzhou, 221000, China
3. Department of Pathology, West China Hospital, Sichuan University, Chengdu, China
4. Department of Medical Oncology, Cancer Center, West China Hospital, Sichuan University, Chengdu, China

* To whom correspondence should be addressed: Dr. Xiawei Wei, PhD, Laboratory of Aging Research and Cancer Drug Target, State Key Laboratory of Biotherapy and Cancer Center, National Clinical Research Center for Geriatrics, West China Hospital, Sichuan University, Chengdu, China. Email: Xiawei Wei (xiaweiwei@scu.edu.cn)

^#^ Xiaoxiao Liu, Ting Yu and Yuzhu Hu contributed equally to this work.

**
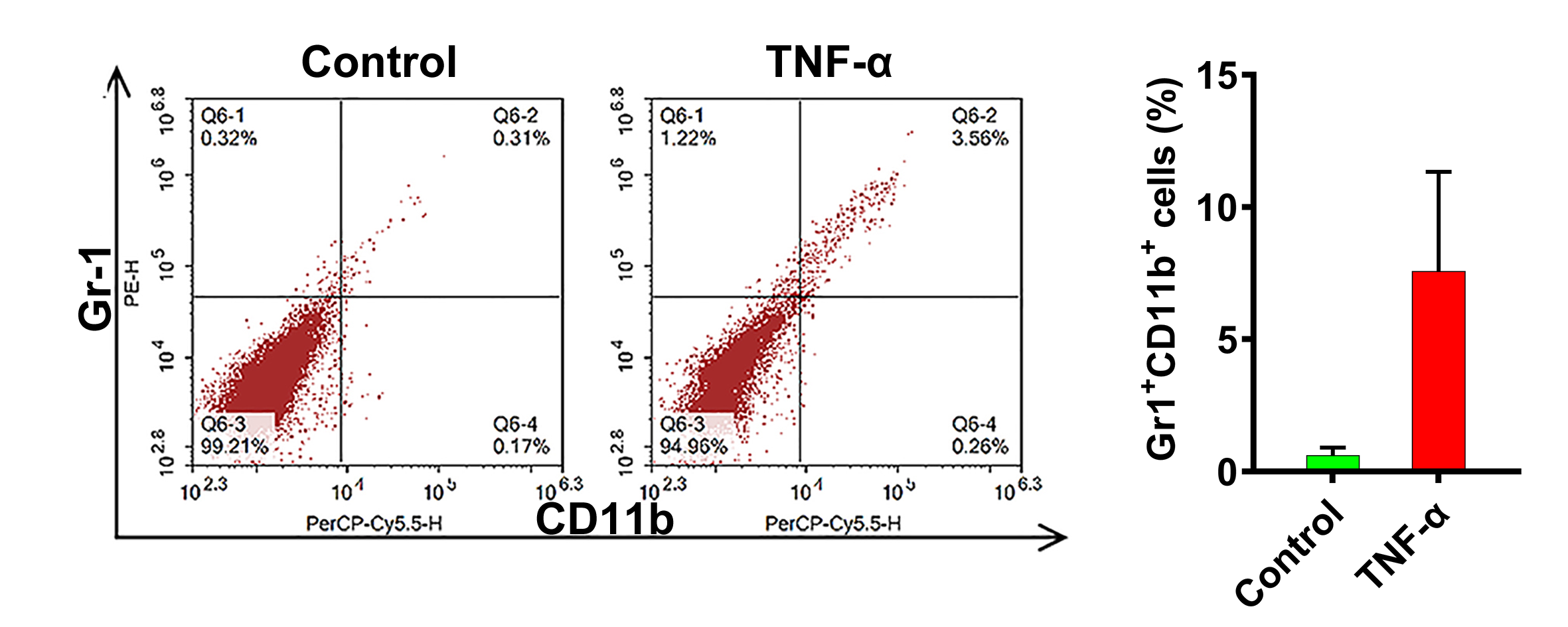
**

**Supplementary Fig. 1** Increased infiltration of Gr1^+^CD11b^+^ cells was detected in TNF-α treated mice in comparison with that in normal mice.

**
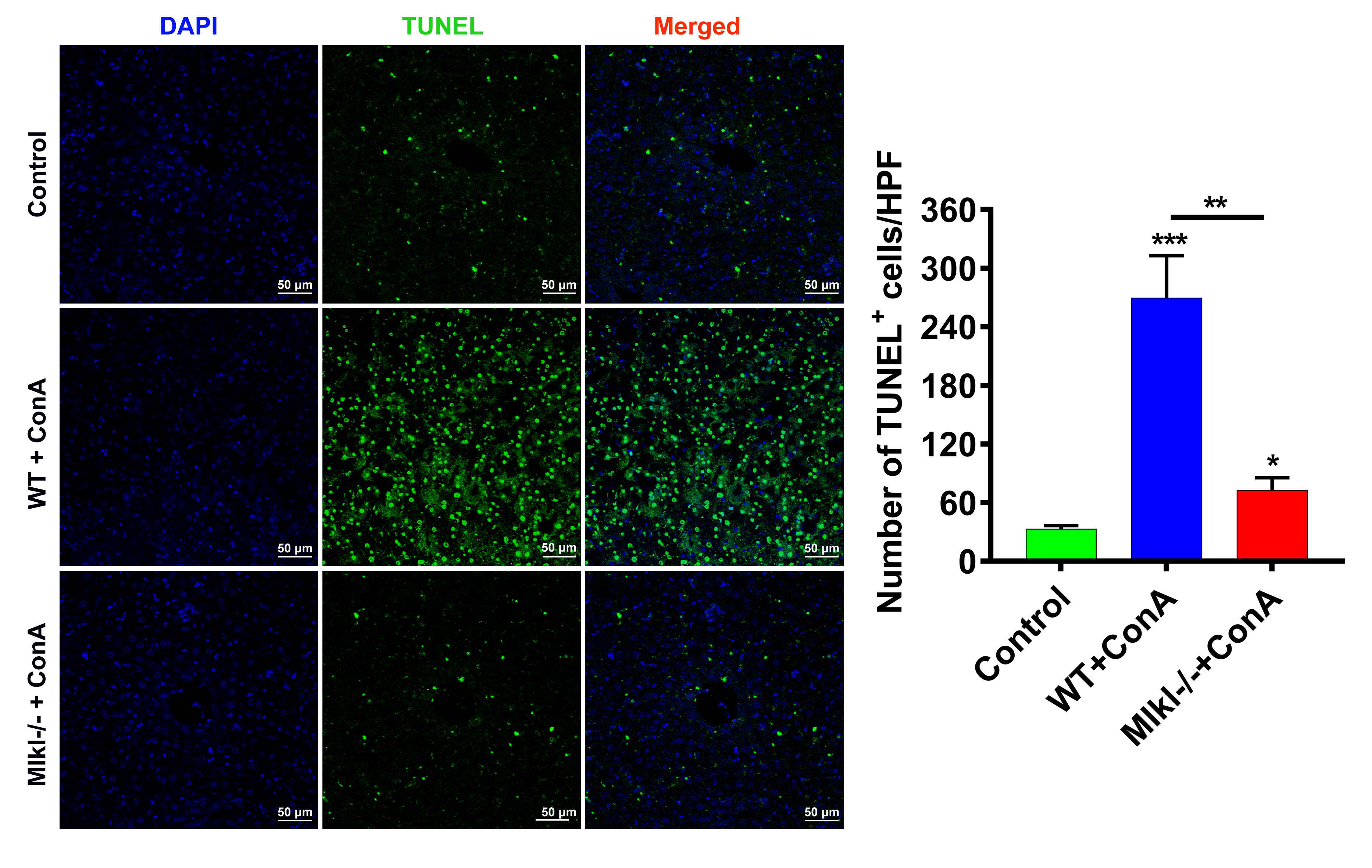
**

**Supplementary Fig. 2** TUNEL staining of liver sections suggested less apoptotic cells in *Mlkl^-/-^* mice than in *WT* mice. (***p < 0.01, ***p < 0.001*).

**
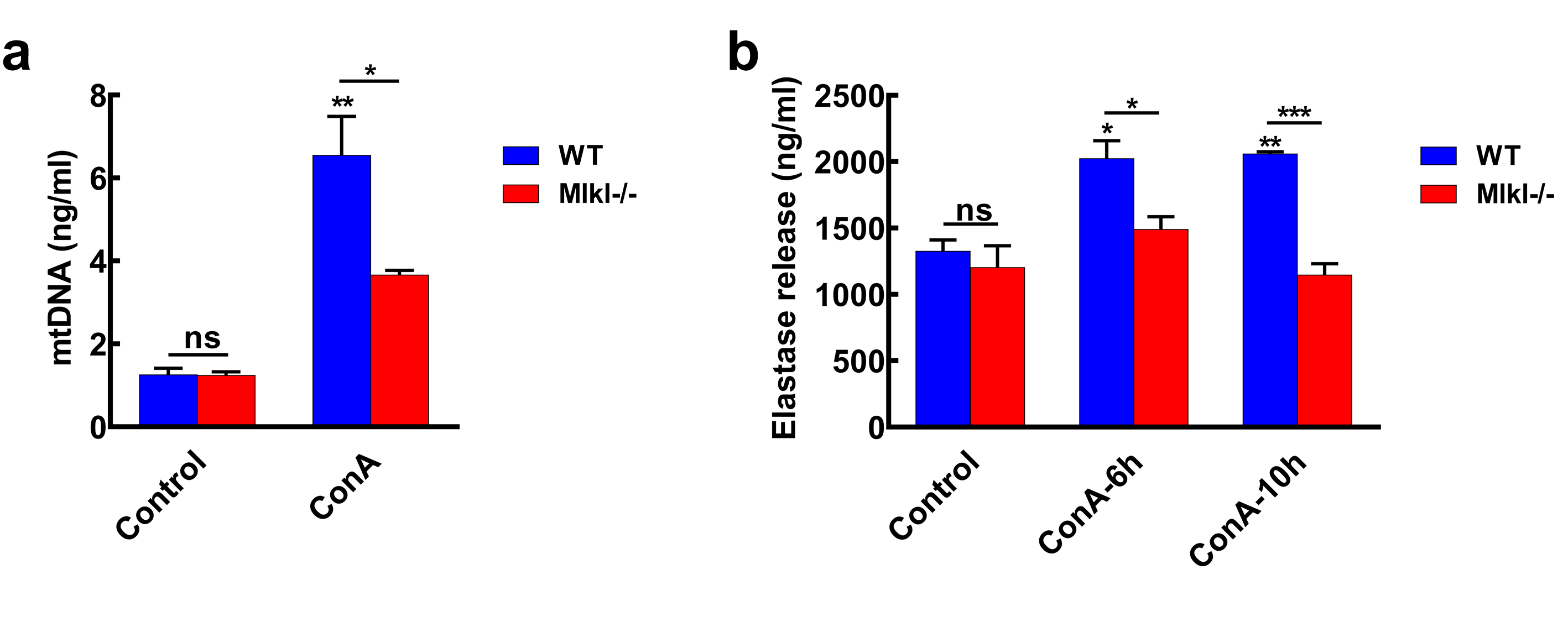
**

**Supplementary Fig. 3 a** The serum level of mtDNA was significantly lower in ConA-treated *Mlkl^-/-^*mice than that in ConA-treated *WT* mice. **b** The ConA treatment significantly promoted the release of elastase in *WT* mice, while no obviously increased elastase was observed in ConA-treated *Mlkl^-/-^*mice. (**p < 0.05, **p < 0.01, ***p < 0.001*).

**
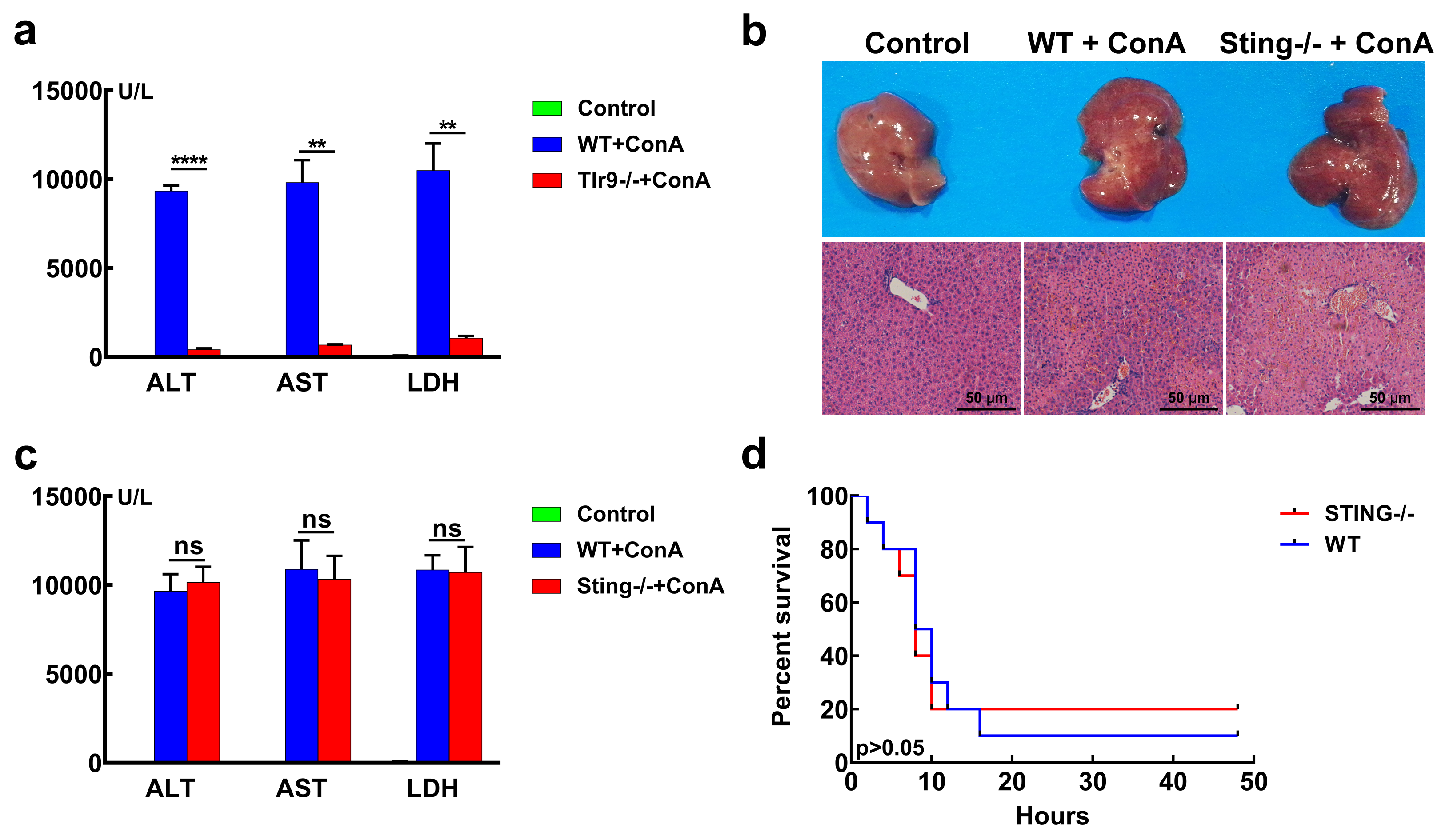
**

**Supplementary Fig. 4 a** The serum levels of ALT, AST and LDH in ConA-treate *Tlr-9^-/-^* mice was significantly lower than that in ConA-treated *WT* mice. **b** No significant difference was found in the gross morphology and the histological morphology of liver between ConA-treated *Sting^-/-^* mice and ConA-treated *WT* mice. **c** No significant difference in the serum levels of ALT, AST and LDH between *Sting^-/-^* mice and *WT* mice after ConA injection. **d** The survival time of ConA-treated *Sting^-/-^* mice was similar to that of ConA-treated *WT* mice. (***p < 0.01, ****p < 0.0001*).
